# Extending the Fundamental Theorem of Biomedical Informatics for the AI era

**DOI:** 10.1093/jamia/ocag065

**Published:** 2026-04-26

**Authors:** Philip R O Payne, Jonathan H Chen, Christopher A Longhurst

**Affiliations:** Institute for Informatics, Data Science, and Biostatistics, Washington University in St. Louis School of Medicine, and Center for Health AI, WashU Medicine and BJC Health, St. Louis, Missouri, United States; Division of Computational Medicine, Division of Hospital Medicine, Clinical Excellence Research Center, and Department of Biomedical Data Science, Stanford University School of Medicine, Stanford, California, United States; Seattle Children’s Hospital and University of Washington School of Medicine, Seattle, Washington, United States

**Keywords:** medical informatics, artificial intelligence, health knowledge, attitudes, practice, systems analysis

## Abstract

**Background:**

Charles Friedman’s Fundamental Theorem of Biomedical Informatics holds that a person working in partnership with an information resource outperforms that same person unassisted. Since its publication, advances in artificial intelligence (AI), adaptive learning systems, and large-scale data infrastructures have transformed the biomedical ecosystem, extending informatics beyond clinical care into domains such as public health, consumer health, translational science, and the broader life sciences. Such expansion has further underscored the importance of the Fundamental Theorem while also elucidating ways it can be expanded to meet current needs.

**Objective:**

To reassess and extend the Fundamental Theorem for the AI era in a manner that preserves its conceptual strength while broadening its applicability across an evolved and more complex biomedical ecosystem.

**Methods:**

This Viewpoint synthesizes empirical evidence and sociotechnical theory related to human-AI collaboration, learning health systems (LHS), learning public health systems (LPHS), AI governance, and systems science to contextualize the Fundamental Theorem within such contemporary frameworks.

**Results:**

We argue that the unit of analysis of the Fundamental Theorem should shift from individuals and tools to adaptive sociotechnical systems spanning clinical care, public health, translational research, consumer engagement, and life sciences innovation. We propose an expanded theorem: A learning biomedical ecosystem that continuously optimizes human-AI collaboration will outperform humans or AI alone.

**Conclusions:**

This evolution builds directly upon Friedman’s original theorem, reaffirming its human-centered foundation, while incorporating AI-enabled computation, adaptive learning, and systems-level integration across the modern biomedical enterprise.

## Introduction: from tools to systems

In 2009, Charles Friedman introduced the Fundamental Theorem of Biomedical Informatics, asserting that a person working in partnership with an information resource is “better” than that same person unassisted.^1^ The theorem intentionally redirected attention away from automation and toward augmentation, positioning Biomedical Informatics as a discipline concerned with empowering human cognition.

Importantly, Friedman’s framing inherently accommodated systems-level thinking. The “information resource” was never merely a tool; it encompassed infrastructures, workflows, and sociotechnical environments. As subsequent commentaries underscore,^2^^,^^3^ the theorem functioned as scaffolding, durable precisely because it could evolve alongside changes in both computational capability and organizational context.^4–7^

Since 2009, the biomedical ecosystem has undergone a dramatic transformation. Generative AI, multimodal learning systems, autonomous agents, and adaptive or continual learning architectures now operate across bioinformatics, translational informatics, clinical informatics, public health informatics, and consumer health informatics.^8–10^ Learning health systems (LHS) are increasingly conceptualized as adaptive, continuously improving infrastructures in which the data generated through care delivery, research, and patient engagement are systematically transformed into knowledge and reintegrated into practice, thereby enabling ongoing optimization of clinical, operational, and translational performance across the health ecosystem.^11^^,^^12^ Similarly, public health systems are increasingly evolving to become scalar learning systems, exemplified by the learning public health system (LPHS) framework, which extends LHS principles to population-level surveillance, preparedness, and policy learning.^13^ Recent scholarship surrounding these frameworks has emphasized the need to integrate AI capabilities into both clinical and public health infrastructures of this type,^14–17^ reinforcing the urgency of systems-level thinking in such contexts.

Based on these developments, we believe that the question is no longer whether a person with a tool outperforms a person alone. Rather, it is about designing adaptive biomedical ecosystems, spanning patients, clinicians, scientists, public health agencies, payers, policymakers, and AI systems, that continuously learn and improve.

## Reframing the Fundamental Theorem

**Figure 1.**
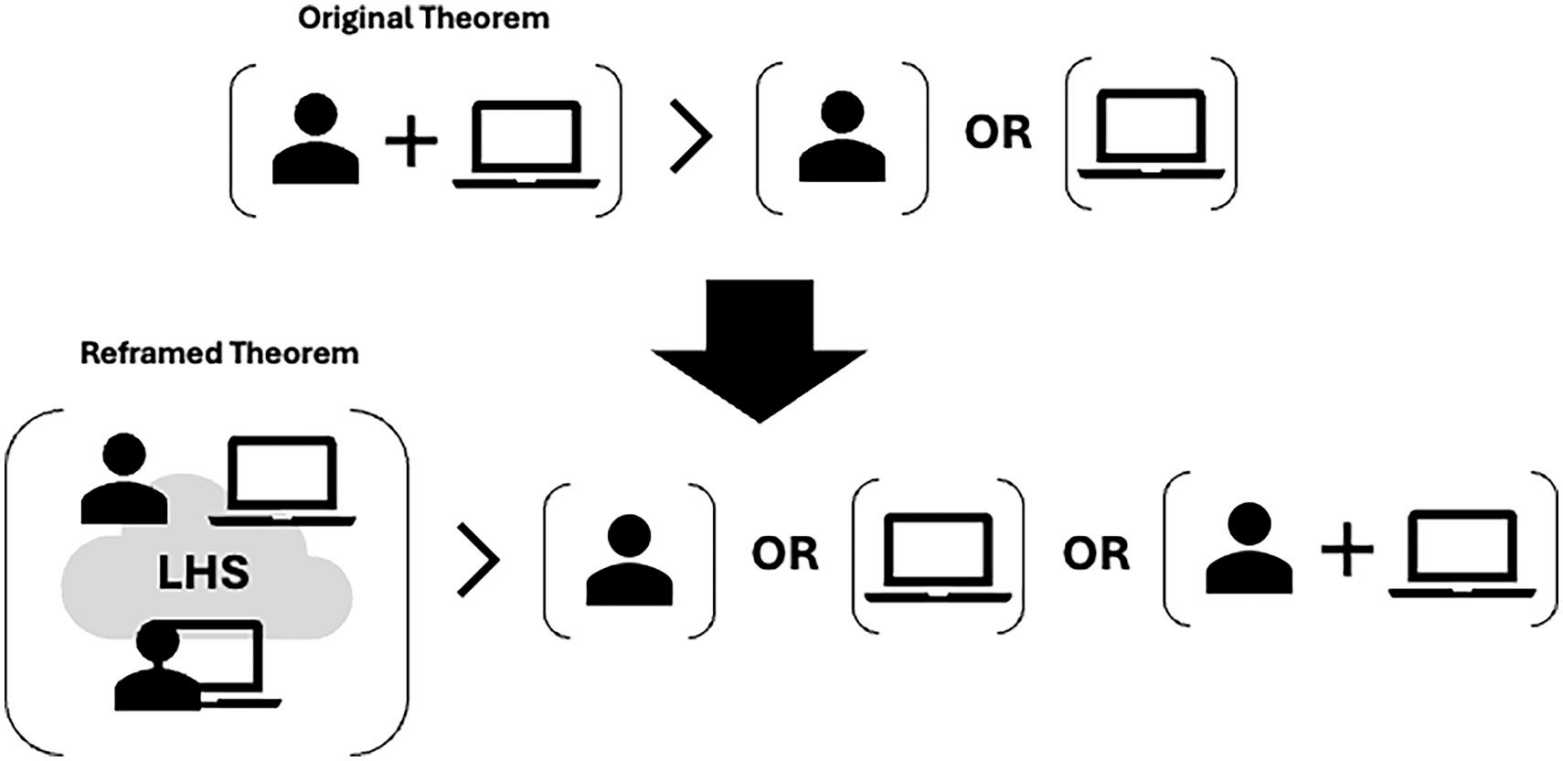
A reframing of the Fundamental Theorem of Biomedical Informatics. The original theorem posits that a person working with an information resource outperforms working alone. The reframed theorem shifts the unit of analysis from individuals and tools to a Learning Health System (LHS) or Learning Public Health System in which humans and computers collaborate dynamically. In this sociotechnical model, the integrated LHS outperforms humans alone, AI alone, or simple human-machine pairings by continuously optimizing cognitive allocation, feedback loops, and governance.

Given the preceding observations, we propose the following extension of Friedman’s Fundamental Theorem (Figure 1), consistent with its graceful evolution, as has been argued for above:

A learning biomedical ecosystem that continuously optimizes human-AI collaboration will outperform humans or AI alone.

This extended formulation builds directly upon Friedman’s original theorem. However, while the original theorem centered on augmentation, the revised formulation expands the locus of augmentation from individuals to interconnected sociotechnical systems. This expansion also reflects the maturation of LHS and LPHS. Further, it recognizes that performance emerges from bidirectional learning loops that span a multitude of domains, including but not limited to life sciences innovation, translational science, clinical care delivery, public health surveillance and response, consumer health and patient engagement, and healthcare policy. Such a view also infers that patients and consumers are not merely recipients of system outputs, but rather, that they increasingly act independently of clinicians through consumer-facing AI tools, wearable technologies, and direct-to-consumer analytics, contributing data and shaping learning loops across the broader health and healthcare ecosystem.

## What is distinctive about the AI era?

Many sociotechnical principles underlying biomedical informatics predate AI, such as human-centered design, workflow integration, cognitive alignment, and system safety.^5^^,^^7^^,^^18–20^ However, what distinguishes the AI era is not the replacement or displacement of these principles, but rather an expansion of computational capability that substantially magnifies their importance. Examples of such developments that define the current AI era include the widespread adoption, evaluation, and use of:

1. *Autonomous and Semi-Autonomous Agents*: AI systems can now initiate actions, triage information, and generate outputs without direct prompting, shifting interaction from passive tool use to active collaboration.^21^
2. *Generative AI*: Large language and multimodal models can synthesize text, images, signals, and structured data, enabling knowledge generation rather than information retrieval.^22^
3. *Adaptive and Continual Learning*: Adaptive and continuous learning technologies can update based on new data, enabling real-time optimization of AI-enabled tasks embedded within LHS and LPHS infrastructures.^23^
4. *Human-AI Teaming*: Using modern AI tools, cognitively complex tasks can be dynamically decomposed and reallocated at the atomic level, enabling a fluid distribution of reasoning between humans and machines.^24^

As an example of the intersection of such developments, we can compare traditional decision support systems that deliver static, rule-based recommendations with AI-enabled decision augmentation that supports probabilistic reasoning, contextual synthesis, adaptive calibration, and interactive generative output. The distinction is not merely technical; it transforms the nature of the partnership between humans and information resources envisioned in the original Fundamental Theorem.

## Cross-ecosystem use cases

To further ground our proposed expansion of Friedman’s Fundamental Theorem, we have considered several illustrative use cases, as follows:

- *AI-supported biomarker and therapeutic agent discovery*: Multimodal AI applied to genomic and phenomic data can accelerate bioinformatics and translational informatics by identifying candidate biomarkers and therapeutic agents, validated through clinical and population-level feedback loops involving both human researchers, clinical collaborators, and large-scale data and knowledge resources.^25^
- *Consumer-facing AI tools*: AI-enabled symptom checkers, wearable sensor analytics, and health coaching systems can allow patients to act independently of clinicians, generating data and influencing care-seeking behavior, while contributing to digitally mediated shared decision-making concerning both health and disease management.^26^
- *Public health learning loops*: AI-enhanced surveillance systems can support outbreak detection, policy adaptation, and the dynamic and iterative planning and assessment of intervention strategies, embodying LPHS principles at the population scale.^27^
- *Payer- and policy-driven learning*: Predictive analytics integrated into reimbursement and coverage models can create feedback loops that link clinical outcomes, cost structures, and policy reform, while expediting point-of-care tasks such as prior authorization and referrals for specialty care.^28^^,^^29^

Of note, each of these examples reflects bidirectional learning across computational and human actors and domains, rather than isolated augmentation.

## Human strengths and computational counterparts

Even with rapid advances in AI technologies, it is important to recognize that humans retain irreplaceable strengths in ethical reasoning, contextual judgment, relational communication, and value alignment.^5^^,^^20^ In contrast, computational systems excel at high-dimensional inference, large-scale pattern recognition, probabilistic modeling, and the consistent synthesis of multimodal data at speeds and scales beyond unaided human cognitive capacity. These differentiations are not competitive; rather, they are complementary, creating the foundation for increasingly sophisticated human-computer partnerships. As noted previously, the AI era enables dynamic allocation of cognitive work, in which human-computer systems can share responsibility for data-intensive, repetitive, or pattern-based tasks while prioritizing the use of unique human cognitive capabilities to interpret and assess complex data, in a manner contextualized by humanistic and moral deliberation. Such rebalancing supports dynamic autonomy, in which oversight and delegation are continuously adjusted in response to task complexity, risk, and outcome-related feedback. Within such a model, patients, clinicians, researchers, and other key decision-makers may each operate independently at times, leveraging AI tools for self-management, decision support, discovery, or surveillance, yet remain embedded within shared learning architectures that integrate their actions into broader systems-level knowledge generation and improvement.

## Risks and safeguards in human-AI collaboration

The expansion of AI-enabled collaboration, such as that contemplated above, does introduce new risks and challenges, several of which are enumerated below:

- *Algorithmovigilance*: The performance of AI tools and technologies can change over time due to both internal and external factors. Therefore, continuous post-deployment monitoring of AI performance across populations is needed to ensure the reproducibility and rigor of model results.^30^
- *Bias amplification*: Reinforcement of inequities through skewed training data or deployment contexts can negatively impact model performance and must be measured and mitigated via systems that account for or otherwise mitigate such issues.^31^
- *Automation bias and overreliance*: Excessive trust in AI outputs at the expense of human judgment can lead to deskilling or equivalent declines in human performance, requiring feedback and learning mechanisms to assess and intervene to prevent such outcomes.^32^^,^^33^

Importantly, LHS and LPHS frameworks can help address these potential pitfalls by embedding continuous monitoring, feedback, recalibration, and governance into operational workflows. In this sense, LHS and LPHS infrastructures function as safeguards for AI deployment, enabling responsible co-evolution and enhanced tracking, monitoring, and due diligence “at scale.”

## Toward a system-level ethos

The challenges and opportunities of the modern AI era must be understood not as a competition between humans and machines, but as a systems-level redesign of how healthcare functions as a complex adaptive enterprise. The core problems facing our healthcare system, persistent quality gaps, preventable harm, uneven outcomes, rising costs, and inequitable access, are not isolated failures of individuals but failures of coordination, information flow, feedback, and alignment across the system. LHS and LPHS frameworks provide the structural scaffolding to address these challenges by integrating data, analytics, clinical workflows, governance, and accountability into continuous improvement cycles. Within this architecture, AI serves not as a replacement for human expertise, but as a force multiplier that redistributes cognitive work across the system. Computational systems can assume responsibility for large-scale data integration, surveillance, prediction, and pattern recognition, enabling earlier risk detection and more consistent adherence to evidence-based practices. Human actors, including clinicians, patients, administrators, and researchers, retain essential roles in contextual interpretation, ethical judgment, prioritization, and relationship-centered care as noted above. Autonomy and oversight are dynamically calibrated according to clinical risk, operational complexity, and societal values. Critically, actions taken at the point of care become inputs into shared learning infrastructures that convert distributed decisions into system-wide knowledge. In this way, human-AI collaboration becomes a mechanism for strengthening reliability, improving outcomes, enhancing value, and expanding access, advancing healthcare not through isolated innovation, but through coordinated, system-level transformation.

## Conclusion

We believe that the proposed extension of the Fundamental Theorem applies across the entire biomedical ecosystem. In doing so, it supports an evolution toward an adaptive biomedical ecosystem, spanning patients, clinicians, scientists, public health agencies, payers, policymakers, and, most recently, AI, that continuously learn and improve together.

Far from supplanting Friedman’s original theorem, this reframing affirms its durability. The original insight, that humans augmented by information resources outperform those unassisted, remains true. What has changed is the scale, autonomy, and adaptive capacity of those resources. In the AI era, the responsibility of biomedical informatics is to design learning biomedical systems in which humans and AI continuously align and co-evolve, advancing equity, safety, scientific discovery, public health, and ultimately, human wellbeing.

## Data Availability

This article is a perspective and does not report or analyze any datasets; therefore, no data are available.

## CRediT

Philip Richard Orrin Payne (Conceptualization, Investigation, Methodology, Writing Original Draft, Writing Review Editing), Jonathan H Chen (Conceptualization, Investigation, Methodology, Writing Original Draft, Writing Review Editing), Christopher Long hurst (Conceptualization, Investigation, Methodology, Writing Original Draft, Writing Review Editing).
